# Vegetation dynamics and its driving force in the Qinghai Lake Basin, China

**DOI:** 10.3389/fpls.2025.1691672

**Published:** 2025-10-21

**Authors:** Jiesheng Sun, Yuanyuan Ding, Yong Wang, Chongyi E

**Affiliations:** ^1^ School of Geographical Sciences, Nanjing University of Information Science and Technology, Nanjing, Jiangsu, China; ^2^ School of Ecology and Applied Meteorology, Nanjing University of Information Science and Technology, Nanjing, Jiangsu, China; ^3^ Key Laboratory of Tibetan Plateau Land Surface Processes and Ecological Conservation (Ministry of Education), Qinghai Normal University, Xining, Qinghai, China

**Keywords:** Qinghai Lake Basin, vegetation cover dynamics, spatiotemporal variation characteristics, driving factors, partial least squares structural equation model

## Abstract

Qinghai Lake Basin is the largest endorheic basin in the northeastern part of the Qinghai-Tibet Plateau (QTP). The vegetation dynamics are subject to dual pressures from climate change and human activities. Previous studies have neglected the interactions among driving factors, as well as the impact of climate factors on vegetation under the regulatory role of topographic elements. The present study utilises MODIS-EVI data from 2001 to 2022 to estimate Fractional Vegetation cover (FVC) and to reveal the spatiotemporal dynamics of vegetation cover through trend analysis and other methods. Furthermore, it elucidates the effect of topographical factors on vegetation distribution. Finally, geographic detectors and the partial least squares structural equation model (PLS-SEM) were employed to quantify the impact intensity of driving factors (including climate, human activities, topography, and soil) and analyze their interactive effects and influence pathways on vegetation cover. The results suggested that (1) FVC in the Qinghai Lake Basin increased significantly (1.38×10^-^³/a); notably, low-grade FVC areas exhibiting high volatility. (2) The terrain effect displays clear differentiation characteristics. FVC peaks in the elevation range of 3500–3800 m, FVC dispersion increased with slope, and semishady/shady slopes dominated FVC distribution. The vegetation improvement type is concentrated on low-elevation, flat slopes and shady slopes, whereas the vegetation degradation type is distributed on middle- and low-elevation slopes and semipositive slopes. (3) Climatic factors primarily exert a direct positive influence on FVC. As far as climate factors are concerned, the effects of temperature and precipitation on FVC do not act independently, but act together through synergistic effects, with temperature showing a more significant driving effect. Topography primarily affects FVC indirectly by regulating water and heat conditions (temperature and precipitation). Each factor possesses an optimal range (elevation: 3400–4100 m, precipitation: 325–550 mm, temperature: −6 to 0°C). When changes in these driving factors exceed the optimal range, FVC is suppressed. On a temporal scale, climate change and human activities are the dominant factors influencing the FVC in the Qinghai Lake Basin. The positive effects of human factors on FVC have strengthened.

## 1 Introduction

The Qinghai-Tibet Plateau, among the region’s most sensitive to global climate change (Liu et al., 2009; Meng et al., 2023), possesses an extremely fragile ecosystem and functions as a crucial ecological barrier and the “Asian water tower” (Immerzeel et al., 2020; Yao et al., 2022). Vegetation serves as the pivotal medium sustaining the hydrological cycle and regulating water resources (Immerzeel et al., 2010). On the one hand, alpine meadows and grasslands anchor soil through their root systems, thereby slowing surface runoff velocity and prolonging precipitation infiltration duration. This facilitates the replenishment of underground aquifers by glacial meltwater and rainfall (Xiao et al., 2024). On the other hand, vegetative transpiration creates a “biological pump”, modulating regional precipitation through land-atmosphere feedback mechanisms (Yan et al., 2025). Vegetation, a key indicator of ecosystem health (Shen et al., 2022), is essential for maintaining carbon-oxygen balance, regulating climate, driving hydrological cycles, and protecting biodiversity (Gerten et al., 2004; Rice et al., 2004; Klynge et al., 2020). Changes in vegetation dynamics indicate the ecosystem’s response to various drivers (LaPaix et al., 2009). Fractional vegetation cover (FVC) is a widely used metric for monitoring vegetation dynamics (Carlson and Ripley, 1997; Li et al., 2024; Liu et al., 2024). Unlike vegetation indices (VI) such as NDVI and EVI, which evaluate vegetation health through greenness, FVC offers a more precise representation of vegetation distribution and coverage (Anees et al., 2024). EVI overcomes the saturation issue of NDVI in high FVC areas through comprehensive atmospheric and soil background correction (Liu and Huete, 1995; Gao, 1996). Therefore, estimating FVC using EVI data better reflects changes in vegetation cover.

The Qinghai-Tibet Plateau, as a alpine mountain region, has complex terrain conditions that regulate the spatial distribution of solar radiation and precipitation, thereby significantly influencing hydrothermal conditions (Li et al., 2021; Xian et al., 2024). As elevation increases, the vertical zonation of vegetation becomes increasingly pronounced, with topographic factors driving the formation of vegetation cover spatial distribution patterns through direct and indirect effects (Fan and Bai, 2021; Pu et al., 2025). Previous studies have investigated the influence of terrain on vegetation cover by analysing the distribution characteristics and trends of the NDVI under different topographic conditions, along with the corresponding area proportions (Wang et al., 2022b; Huang et al., 2023). However, Zou et al. (2025) explored the global alpine zone and found that with increasing elevation, the positive trend magnitude of vegetation greenness decreases, while that of vegetated areal fraction increases in most regions (85.49%). Furthermore, Slope aspect impacts alpine vegetation changes globally, with distinct differences in vegetation greenness and vegetated areal fraction trends across aspects (Wang et al., 2022d; Huang et al., 2023). Therefore, relying solely on a single vegetation index is insufficient to comprehensively reveal the comprehensive effects of topography on vegetation cover. For example, although the actual area of vegetation change under certain terrain conditions may be small, its proportion of the total area of vegetation change under all terrain conditions may be high, which can lead to uncertainty in the assessment of the impact of terrain factors on vegetation change. By introducing a terrain distribution index (Wang et al., 2023b), the influence of differences in the absolute areas of different terrain factors can be eliminated, revealing the impact of specific terrain factors on vegetation cover change and clarifying the influence of different terrain conditions on the distribution of vegetation change types and their evolutionary trends.

The Qinghai Lake Basin, the largest endorheic basin in the northeastern Qinghai-Tibet Plateau, is an alpine semi-arid mountainous region that is highly sensitive to global climate change (Aldwaik and Pontius, 2012; Cao et al., 2025). In recent decades, the basin’s ecosystem has faced dual pressures from climate warming and intensified human activities (Qin and Huang, 1998; Dong et al., 2018; Zhang et al., 2025). Since the 1950s, the region has experienced grassland degradation and aridification, with the vegetation growth environment continuing to deteriorate (Shang and Long, 2007; Li et al., 2013; Wang et al., 2022c). However, since 2005, the implementation of ecological restoration projects has led to significant improvements in grassland vegetation conditions, undergoing a complex process of degradation and recovery (Dong et al., 2020). In previous studies, Guo et al. (2014) analysed vegetation cover changes in the Qinghai Lake basin from 2001 to 2012 using MODIS-EVI data, finding that 77.90% of the area showed an upward trend in EVI. The areas with significant improvements were primarily concentrated along the southern shore of Qinghai Lake and in the central part of the basin, while areas of vegetation degradation were concentrated in the eastern part of the basin; Xuelu et al. (2016) used average NDVI during the growing season to analyse vegetation cover changes from 2001 to 2010, finding that areas with improved vegetation were mainly concentrated in the Buha River basin and the central region north of Qinghai Lake, while localized vegetation degradation was observed along the shores of Qinghai Lake. Both studies confirm the overall trend of vegetation improvement in the Qinghai Lake basin, though differences in the areas showing improvement or degradation may arise due to variations in remote sensing data and the temporal scale of the studies. Vegetation dynamics result from the interplay of natural factors and human activities (Liu et al., 2019b; Gu et al., 2022), including climate change, topography, soil, and human activities (such as grazing and land-use changes) (Berdugo et al., 2022; Gao et al., 2022; Kolluru et al., 2022; Liu et al., 2023). While climate factors are widely recognized as primary drivers (Liu et al., 2021a; Xu et al., 2024), the exact contributions of other influences remain unquantified. These drivers do not act independently on vegetation; their interaction mechanisms and the pathways through which they influence vegetation dynamics remain unclear. In addition, traditional residual statistics methods lack real data verification to distinguish between the effects of climate change and human activities on vegetation dynamics (Zhang and Ye, 2021).

Current research on vegetation dynamics in the Qinghai Lake basin lacks long-term remote sensing data monitoring, and the contributions of natural factors (such as climate change, topography, and soil) and human influences on FVC remain underexplored. The study aims to strengthen understanding of vegetation dynamics and driving mechanisms in the Qinghai Lake basin through three objectives: (1) Employing the Sen slope estimator and coefficient of variation to characterize the spatiotemporal patterns and stability of FVC, and using a transition matrix to capture spatiotemporal changes in FVC across different grades; (2) Utilizing a terrain distribution index to examine spatial patterns of FVC changes under varying terrain conditions and to assess FVC distribution characteristics; (3) Quantifying the impact of human activities on vegetation via land use and resolution grazing intensity data, and integrating the geographic detector model with the Partial least squares structural equation modeling (PLS-SEM) to analyze the contributions of various driving factors to FVC distribution and their mechanisms on FVC’s spatiotemporal changes. The findings offer valuable insights for ecological conservation in high-elevation semi-arid ecosystems.

## 2 Materials and methods

### 2.1 Study area

**Figure 1:**
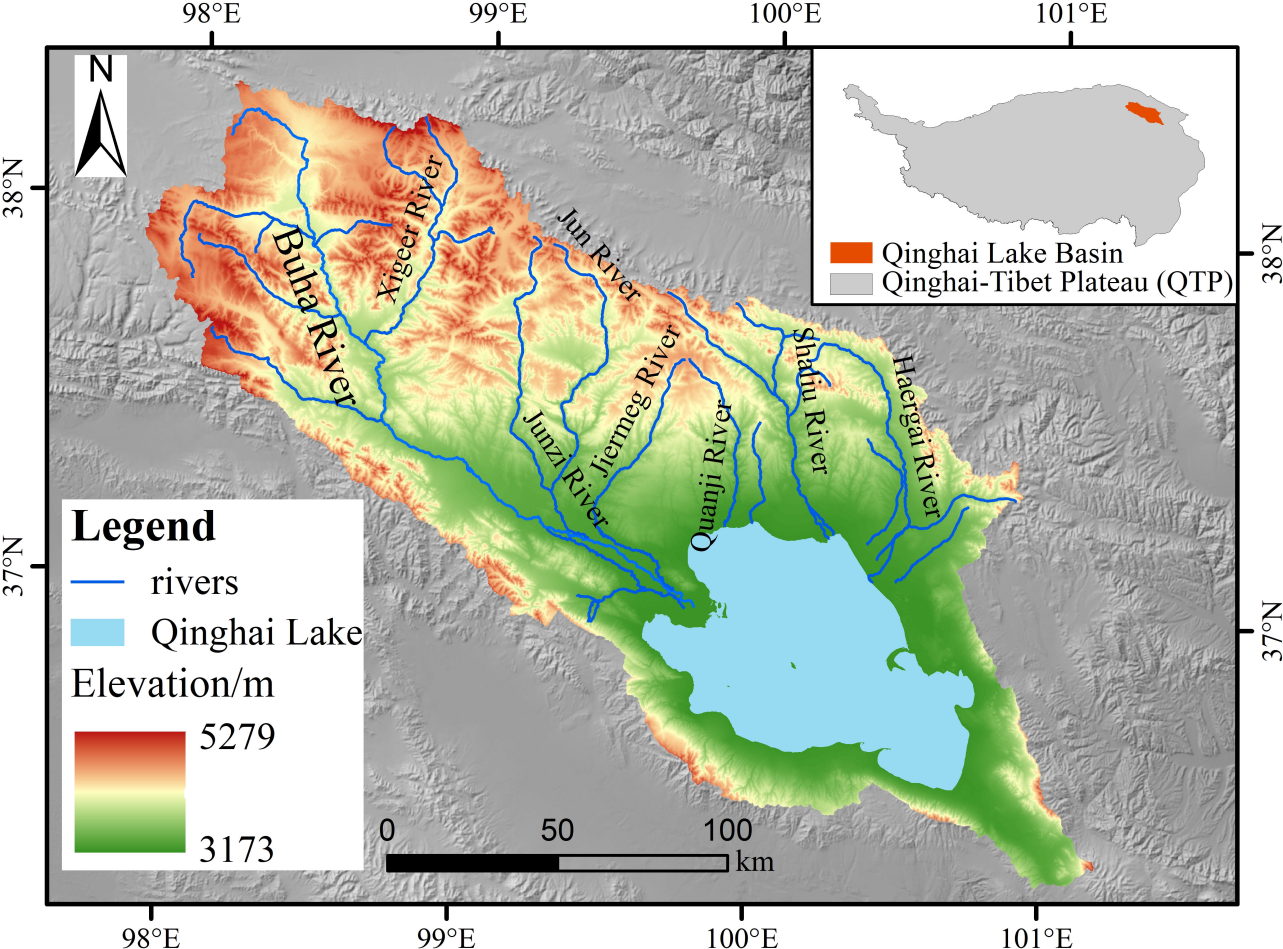
The geographic situation of the Qinghai Lake Basin.

The Qinghai Lake Basin, the largest endorheic basin in the northeastern Tibetan Plateau (36°15’N-38°20’N, 97°50’E-101°20’E), spans elevations from 3,173 to 5,279 m. Its topography is characterized by high terrain in the northwest, lower areas in the southeast, and a central lowland, forming a closed plateau basin surrounded by mountains (**Figure 1**). Covering 29,661 km² (Wang et al., 2025), this basin lies at the intersection of China’s eastern monsoon, southwestern alpine, and northwestern arid zones, featuring a semiarid alpine climate with an average temperature of 0.7 °C and annual precipitation of 404.1 mm (Zhang et al., 2024). Qinghai Lake, China’s largest inland saltwater lake (Ma et al., 2024b), is situated in the basin’s southeast and is fed by about 50 rivers. The Qinghai Lake Basin comprises eight subbasins, with the Buha River Basin being the largest, covering roughly 50% of the total basin area (Zhang et al., 2014).

### 2.2 Data sources

In this study, we utilized MODIS data (MOD13Q1), specifically the 16-day synthetic EVI index with a 250 m spatial resolution, enhancing sensitivity in high-biomass regions. This data, acquired from NASA, was preprocessed using ENVI software through splicing, projection, and format conversion. The maximum value composite (MVC) method was employed to mitigate the effects of clouds, atmospheric conditions, and solar elevation, producing monthly EVI data. These monthly figures were then averaged to derive annual EVI data.

Vegetation growth is shaped by natural and anthropogenic factors (Wang et al., 2023a; Zhao et al., 2024). This study considered climatic variables (temperature and precipitation), topographic features (elevation, slope, and aspect), soil properties (soil type and organic carbon content), and anthropogenic factors (land use, population density, and high-resolution grazing intensity). To maintain consistency in spatial analysis and PLS-SEM model construction, temperature, precipitation and grazing intensity data were processed using inverse distance weighting (IDW) (Lu and Wong, 2008). Digital Elevation Model (DEM) data underwent bilinear interpolation (Xu et al., 2016), while land use data employed nearest neighbour interpolation to preserve land attribute characteristics. Data sources are detailed in **Table 1**. All data were resampled in ArcGIS to a spatial resolution of 250 m (Guo et al., 2024).

**Table 1:** Sources of data.

Data type	Factors	Abridge	Resolution	Source	Time periods
Topography	Slope	Slp	90m	–	–
Topography	Slope direction	Asp	90m	–	–
Topography	Elevation	Ele	90m	Geospatial data cloud (http://www.gscloud.cn/); National Tibetan Plateau Science Data Center (https://npdc.ac.cn/)	2000
Climate	Temperature	Tmp	1 km	National Tibetan Plateau Science Data Center (https://data.tpdc.ac.cn/)	2001 - 2022
Climate	Precipitation	Pre	1 km	National Tibetan Plateau Science Data Center (https://data.tpdc.ac.cn/)	2001 - 2022
Human Activity	Land cover	LUCC	30m	National Cryosphere Desert Data Center(https://data.tpdc.ac.cn/)	2001 - 2022
	long - term High - resolution Grazing Intensity	LHGI	250m	National Ecosystem Science Data Center(http://nesdc.org.cn)	2001 - 2022
Human Activity	Population density	Popd	1 km	Land Scan Global Population Data(https://landscan.ornl.gov) and International Soil Reference and Information Centre (https://data.isric.org)	2001 - 2022
Soil	Soil type	Soilt	–	Harmonized World Soil Database (HWSD) (https://data.tpdc.ac.cn/)	2000
Soil	Soil organic carbon	SOC	250m	Harmonized World Soil Database (HWSD) (https://data.tpdc.ac.cn/)	2000

### 2.3 Study framework

**Figure 2:**
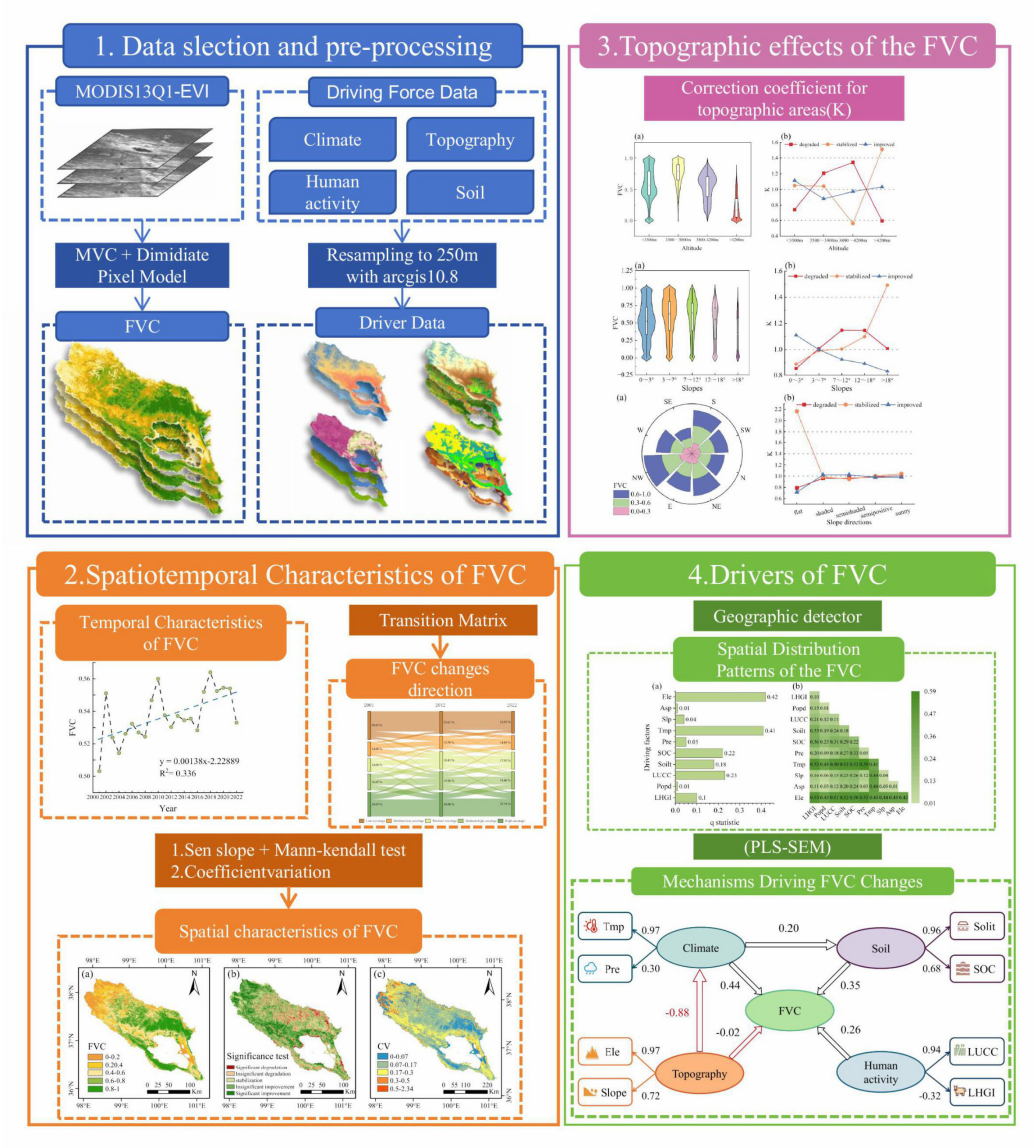
Research process.

The present study was divided into four stages (**Figure 2**). FVC was estimated to use the MVC and the Dimidiate Pixel Model based on MODIS-EVI, in addition we calculated the average of the individual driver variables. Subsequently, the Theil-Sen median trend analysis and Mann-Kendall Test were employed to calculate the trend of FVC from 2001 to 2022, and Coefficient of variation (CV) was calculated to assess the volatility of FVC changes. Finally, we analyzed FVC distribution characteristics under varying conditions, employing terrain distribution index for topographic areas to assess the dominant distribution of FVC changes. The Geographic Detector quantified each driver’s impact on FVC’s spatial distribution, and PLS-SEM model was used to examine FVC’s driving mechanisms across temporal and spatial scales.

When conducting drive analysis using geographic detectors, spatial discretization of continuous variable data is required. Based on previous research, natural breakpoint methods are employed to discretize data such as temperature, precipitation, and elevation (Wang et al., 2023d; Yu et al., 2025). When constructing the PLS-SEM model, all raw data undergoes standardized processing to eliminate units of measurement, facilitating comparisons between variables (Shi et al., 2024). Furthermore, based on the results from the geodetector analysis, certain factors were excluded from the PLS-SEM model construction due to their insufficient spatial explanatory power (MA et al., 2024a).

### 2.4 Dimidiate pixel model

The dimidiate pixel model is a widely used approach for estimating FVC (Leprieur et al., 1994). It is based on the premise that the spectral data in a pixel is divided into two components: soil and vegetation. Thus, EVI is derived from the two components of soil and vegetation, and its calculation formula can be expressed as Equation 1:


(1) $$\begin{array}{c} EVI=EVI_{soil}+EVI_{veg} \end{array}$$

where $EVI_{soil}$ represents the pixel value of pure soil and $EVI_{veg}$ represents the pixel value of pure vegetation. The binary pixel model is not restricted by geographical conditions, is easy to promote and use, and can reduce the influence of atmospheric and soil backgrounds to a certain extent. The annual average EVI is calculated via pixel-by-pixel calculations (Equation 2):


(2) $$\begin{array}{c} \bar{EVI}=\frac{\sum_{i=1}^{n}EVI}{n} \end{array}$$

where n is the year number and $\bar{EVI}$ is the average value.

The EVI is used to calculate the FVC as follows (Equation 3):


(3) $$\begin{array}{c} FVC=\frac{EVI-EVI_{soil}}{EVI_{veg}-EVI_{soil}} \end{array}$$

In theory, $EVI_{soil}$ should be approximately zero in most cases. However, due to differences in surface environmental conditions across regions, it varies over time and space, with a typical range of -0.1 to 0.2. Similarly, it also changes with variations in vegetation type and season. Therefore, a $EVI_{soil}$ and $EVI_{veg}$ values are clearly inaccurate. Thus, a 0.5% confidence level is selected, meaning that pixels with a cumulative percentage of 0.5% are pure soil pixels, and those with 99.5% are pure vegetation pixels. The corresponding EVI values for these $EVI_{soil}$ and $EVI_{veg}$ , respectively.

### 2.5 Maximum value composite

The maximum value composite (MVC) method is the most prevalent international approach for maximizing composites. It effectively reduces partial interferences from clouds, atmospheric conditions, and solar elevation angles in monthly EVI data (Li et al., 2019). Utilizing the MVC method, the annual maximum EVI value is determined, enabling a more accurate reflection of surface vegetation cover (Equation 4).


(4) $$\begin{array}{c} MEVI_{ij}=MAX\left(EVI_{ij}\right) \end{array}$$

where i is the year number, j is the month number, and $EVI_{ij}$ is the maximum EVI value for the month of the year.

### 2.6 Trend analysis

The Theil–Sen median trend analysis (Sen, 1968; Theil, 1992) is a nonparametric statistical method for assessing interannual changes in vegetation cover (Zuo et al., 2022). This method is highly efficient and resistant to interference, and it can be expressed as Equation 5.


(5) $$\begin{array}{c} \rho =median\;\frac{x_{j}-x_{i}}{j-i}\;1<i<j<n \end{array}$$

where n is the number of years studied, $x_{i}$ and $x_{j}$ are time series data, and is the trend degree. When ρ < 0, the time series shows a downward trend, and when ρ > 0, the time series shows an upward trend.

The Mann-Kendall Test, a nonparametric method introduced by Mann and Kendall, evaluates time series trends (Equationd 6). Uninfluenced by sample values or distribution types, it effectively analyzes overall data trends and is extensively applied in trend analysis of non-normally distributed data (Fensholt et al., 2009).


(6) $$\begin{array}{c} S=\sum_{i=2}^{n}\sum_{j=1}^{i-1}sign\left(x_{i}-x_{j}\right) \end{array}$$

where x is the time series data, n is the number of data samples, $x_{i}$ and $x_{j}$ are the data values corresponding to years i and j , respectively, where i < j , and the sign is the sign function, with the following rules (Equation 7):


(7) $$\begin{array}{c} sign\left(x_{i}-x_{j}\right)=\{\begin{array}{c} 1\;\;\;\;x_{i}-x_{j}>0\\ 0\;\;\;\;x_{i}-x_{j}=0\\ -1\;\;\;x_{i}-x_{j}<0 \end{array} \end{array}$$

When n >0, the standard normal distribution statistic Z is calculated as follows (Equation 8):


(8) $$\begin{array}{c} Z=\{\begin{array}{c} \frac{S-1}{\sqrt{Var\left(S\right)}}\;\;\;\;S>0\\ \;\;\;0\;\;\;\;\;\;\;S=0\\ \frac{S+1}{\sqrt{Var\left(S\right)}}\;\;\;\;S<0 \end{array} \end{array}$$

The variance formula is as follows (Equation 9):


(9) $$\begin{array}{c} Var\left(S\right)=\frac{n\left(n-1\right)\left(2n+5\right)}{18} \end{array}$$

where n is the number of sample data items.

Based on ρ and Z in trend analysis, we categorize spatial variations in FVC into five types: significant improvement ( ρ ≥0.0005, |Z| ≥1.96), insignificant improvement ( ρ ≥0.0005, |Z| <1.96), stabilization ( |ρ| <0.0005, |Z| <1.96), insignificant degradation ( ρ ≤-0.0005, |Z| <1.96), and significant degradation ( ρ ≤-0.0005, |Z| ≥1.96).

### 2.7 Coefficient of variation

The coefficient of variation is used to indicate the degree of fluctuation in geographic data (Equation 10) and can be used to a certain extent to reflect grassland growth conditions (Tucker et al., 1991; Mao et al., 2022):


(10) $$\begin{array}{c} CV_{FVC}=\frac{1}{x}\sqrt{\frac{1}{\left(n-1\right)}\sum_{i-1}^{n}{\left(X_{i}-\bar{X}\right)}^{2}} \end{array}$$

where $CV_{FVC}$ is the coefficient of variation of the FVC ; i is the time series; $X_{i}$ is the FVC in year i ; and $\bar{X}$ is the average FVC from 2001 to 2022.

### 2.8 Transition matrix

The FVC transition matrix effectively tracks the dynamics of mutual conversions among various vegetation cover types within the study area (Aldwaik and Pontius, 2012). It reveals the transition direction for each vegetation cover type and specifies the conversion area for each type (Equation 11).


(11) $$\begin{array}{c} s_{ij}=\left[\begin{array}{ccc} s_{11} & \cdots & s_{1n}\\ \vdots & \ddots & \vdots \\ s_{n1} & \cdots & s_{nn} \end{array}\right]\; \end{array}$$

where i and j are the FVC grades studied throughout the study period; n is the total number of FVC grades; and $s_{ij}$ is the total area converted from grade i to grade j during the study period.

### 2.9 Terrain distribution index

To assess the influence of terrain on vegetation cover changes, localized changes in specific terrains can appear disproportionately large relative to their overall presence in the study area, potentially skewing evaluations. To accurately determine the impact of terrain factors on vegetation distribution changes, terrain area difference correction is required (Wang et al., 2023b). The terrain area correction coefficient is calculated using the following formula (Equation 12):


(12) $$\begin{array}{c} k=\frac{S_{ie}}{S_{e}}/\frac{S_{i}}{S} \end{array}$$

where $S_{ie}$ is the area of type i changes in terrain $S_{e}$ ; $S_{i}$ refers to the total area of type i changes; $S_{e}$ is the total area of terrain $S_{e}$ ; S is the total area of the study area; $S_{ie}$ / $S_{e}$ is the ratio of the area of type i changes in terrain e ; and $S_{i}$ is the ratio of the area of type i changes in the study area. If k >1, it indicates that type i changes are dominantly distributed in terrain e ; if k =1, it indicates that type i changes are evenly distributed in terrain e ; if k <1, it indicates that type i changes are not dominantly distributed in terrain e .

### 2.10 Geographic detector

Utilizing geodetector to identify spatial variations in the FVC and uncover the drivers of its spatial distribution hinges on the premise that a significant influence of an independent variable on a dependent variable (Wang et al., 2025) implies similar spatial distributions for both. This method involves analyzing variance within and between layers, calculating the single-factor q value, and the q value after superimposing multiple factors (Equation 13), to assess interactions and relationships between factors (Tao et al., 2022; Zuo et al., 2022).

Factor detection assesses the influence of driving factors on FVC, with larger values indicating stronger spatial explanatory power. Interaction detection evaluates the impact of interactions between two driving factors on FVC, determining whether the effect is enhanced, weakened, or independent (Li et al., 2022; Lou et al., 2023; Zhao et al., 2024). First, calculate the q-values for the two individual factors Xn and Xm, as well as the q-value for the two-factor interaction. By comparing q(Xn), q(Xm), and q(Xn⋂Xm), determine the type of interaction between the two driving factors on FVC (**Table 2**). This study implements the Geodetector method using the GD package in R.

**Table 2:** Types of interaction.

Interaction relationship	Interaction types
*q*(Xn⋂Xm)< Min(*q*(Xm), *q*(Xn))	Nonlinear-weaken
Min(*q*(Xn), *q*(Xm))< *q*(Xn⋂Xm)< Max(*q*(Xn), *q*(Xm))	Uni-variable weaken
*q*(Xn⋂Xm) > Max(*q*(Xn), *q*(Xm))	Bivariable enhanced
*q*(Xn⋂Xm) = *q*(Xn) + *q*(Xm)	Independent
*q*(Xn⋂Xm) > *q*(Xn) + *q*(Xm)	Nonlinear enhanced

‘⋂’ denotes the interaction between factors Xn and Xm.


(13) $$\begin{array}{c} q=1-\frac{1}{N\sigma^{2}}\sum_{n=1}^{L}N_{h}\sigma_{h}^{2} \end{array}$$

Where q is the spatial heterogeneity of a certain indicator, N is the total number of samples in the study area, $\sigma^{2}$ is the variance of the indicator, h is the number of partitions, and L is the number of partitions of variables or independent variables. The value of q reflects the degree of spatial differentiation, with a range of [0, 1]. The larger the q value is, stronger the spatial heterogeneity. When q = 0, it indicates that is no spatial heterogeneity.

### 2.11 Partial least squares structural equation model

A structural equation model was developed to examine the influence of both natural variables (climate, topography, and soil) and human-induced factors on FVC changes. Partial least squares structural equation modeling (PLS-SEM) was employed to analyze these systemic relationships (Pearl, 2012; Sarstedt et al., 2022). This approach involves measurement and structural equations. Measurement equations describe the links between indicators and latent variables, while structural equations define the interactions among latent variables. The measurement equations are formulated as follows (Equation 14):


(14) $$\begin{array}{l} X=\Lambda_{x}\xi +\delta \\ \begin{array}{c} Y=\Lambda_{Y}\eta +\epsilon \end{array} \end{array}$$

where X and Y are vectors of exogenous and endogenous indices, respectively, and vectors of exogenous and endogenous latent variables. The corresponding parameter requires estimation, and the disturbance term is included. The structural equation is (Equation 15):


(15) $$\begin{array}{c} \eta =B\eta +\Gamma \xi +\zeta \end{array}$$

B represents the relationship between endogenous latent variables, Γ denotes the influence of exogenous latent variables on endogenous latent variables, and ζ is the error term.

This study employed the “plspm” package in R software to construct the model, the performance of which was assessed through the coefficient of determination (R^2^), R^2^ > 0.67 indicates that the observed variables possess strong explanatory power for the latent variables (Sarstedt et al., 2022) and goodness-of-fit (GOF) metrics, GOF > 0.36 indicates a strong degree of model fit (Wetzels and Odekerken, 2009; Krämer and Sugiyama, 2011; Henseler and Sarstedt, 2013) (**Table 3**).

**Table 3:** PLS-SEM model evaluation criteria.

Criteria	Value	Description
R^2^	> 0.67	Substantial explanatory power
R^2^	> 0.33	Moderate explanatory power
R^2^	> 0.19	Weak explanatory power
GOF	> 0.1	weak model fitting
GOF	> 0.25	medium model fitting
GOF	> 0.36	strong model fitting

## 3 Results and analysis

### 3.1 Characteristics of the spatial and temporal variations of FVC

#### 3.1.1 Temporal changes in the FVC

**Figure 3:**
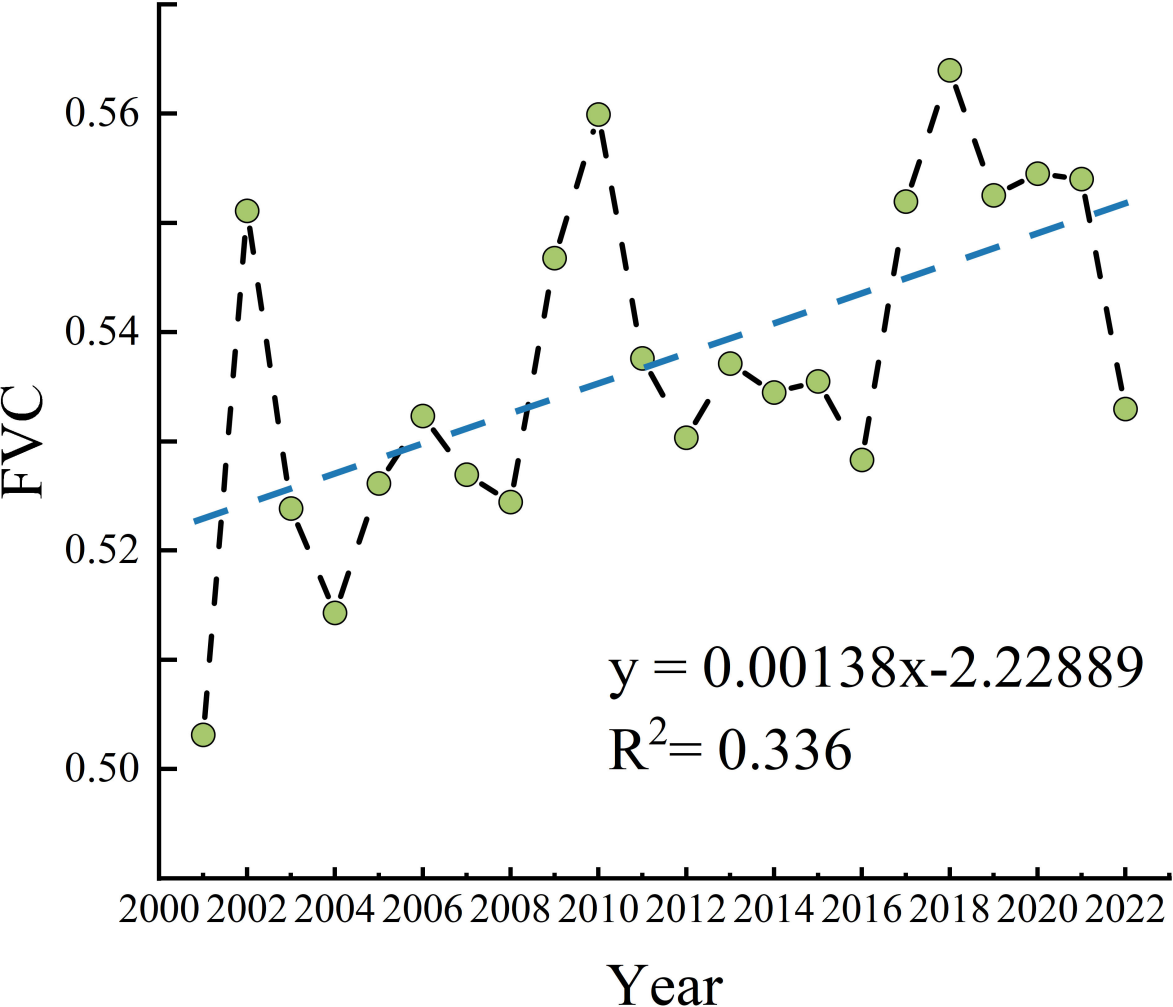
Temporal trends in the FVC from 2001 to 2022. (The green dots represent the mean FVC for each year; the black line denotes the interannual variation in FVC; and the blue dashed line indicates the linear trend of this variation).

The FVC in the Qinghai Lake Basin fluctuated between 0.50 and 0.57 from 2001 to 2022 (**Figure 3**), with a multiyear FVC mean value of 0.53, in which the maximum value of the FVC appeared in 2018, the minimum value of the FVC appeared in 2001, and the basin’s annual average FVC generally showed a fluctuating and increasing trend. (Slope = 1.38 × 10^−3^/a, p<0.005).

#### 3.1.2 Spatial variation in the FVC

**Figure 4:**
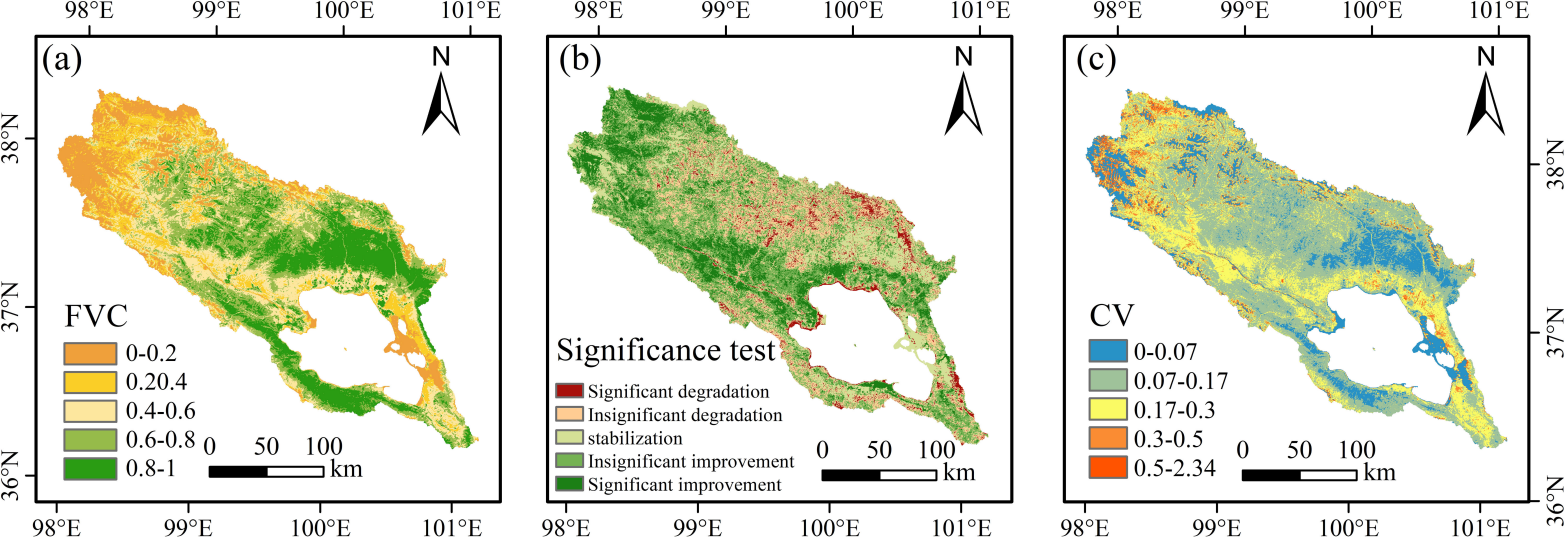
Spatial variation in the FVC from 2001 to 2022. **(A)** average FVC, **(B)** the trend types of the FVC, **(C)** the coefficient of variation (CV) of FVC.

The study categorizes FVC into five grades: low coverage (0.00-0.20), medium-low coverage (0.20-0.40), medium coverage (0.40-0.60), medium-high coverage (0.60-0.80), and high coverage (0.80-1.00). The spatial distribution of FVC in the Qinghai Lake Basin from 2001 to 2022 (**Figure 4A**) reveals temporal changes in the multi-year average FVC. Low and medium-low grade FVC areas, covering 30.83% of the region, are mainly found in the basin’s western part and along Qinghai Lake’s eastern shore. In comparison, medium-high and high-grade FVC areas accounted for 46.15% of the total area (**Table 4**). Larger units were predominantly found in the central region of the basin, whereas smaller units were distributed in a narrow, elongated, band-like formation extending north-west from the south-western shore of Qinghai Lake. **Figure 4B** depicts the FVC trend in the Qinghai Lake Basin during this period. Significant changes affect 23.4% of the basin, with 18.02% primarily occurring in the basin’s northwest and the Buha River’s middle and lower reaches.

**Table 4:** Areas of different FVC grades and their percentages.

Grading standard	Vegetation classification	Area/km^2^	Percentage/%
0.00-0.20	Low coverage	3938.13	15.53
0.20-0.40	Medium-low coverage	3876.75	15.29
0.40-0.60	Medium coverage	5836.00	23.02
0.60-0.80	Medium-high coverage	6685.63	26.37
0.80-1.00	High coverage	5013.88	19.78

Significantly degraded areas account for 5.40% of the total area, primarily concentrated in the central region basin north of Qinghai Lake, while scattered occurrences are observed along the eastern shore of Qinghai Lake, south of the lake, and in the southeastern part of the basin. Area statistics for the five FVC types vary across different FVC levels (**Table 5**). The data reveals that areas with notable improvement in low and medium-low FVC constitute the largest shares (23.49% and 24.12%, respectively). Conversely, high-grade FVC areas are mostly stable (32.77%), indicating that significant improvements are mainly in low and medium-low FVC regions. Notably, areas with significant degradation are predominantly those with medium to high FVC levels, with high-grade FVC areas comprising 66.62% of total significant degradation. This suggests that despite overall watershed improvement, degradation persists in high-grade FVC regions.

**Table 5:** Areas of different types of changes in the FVC and their shares.

Slope	Z	Change type	Area/km^2^	Percentage/%
≥0.0005	≤-1.96	Significant degradation	1369.94	5.40
≥0.0005	-1.96-1.96	Insignificant degradation	5573.06	21.98
-0.0005-0.0005	-1.96-1.96	stabilization	4640.31	18.30
≤-0.0005	-1.96-1.96	Insignificant improvement	9197.62	36.28
≤-0.0005	≥1.96	Significant improvement	4569.31	18.02

The coefficient of variation (CV) for interannual FVC changes ranged from 0.01 to 2.34, with an average of 0.16, demonstrating significant spatial variability (**Figure 4C**). The study categorises the degree of fluctuation in FVC changes into five classes: Stabilisation (CV<0.07), Slight Fluctuation (CV:0.07-0.17) Moderate Fluctuation (CV:0.17-0.30), High Fluctuation (CV:0.30-0.50) and Wild Fluctuation (CV>0.50). In the central basin and the regions south and east of Qinghai Lake, FVC fluctuations diminished, indicating enhanced stability. In contrast, the northwestern basin and the riparian areas of the Habu River basin exhibited heightened FVC variability and reduced stability. Analysis of FVC stability types and their proportions (**Table 6**) indicated that stable and high-volatile types coexisted in low-grade FVC areas, with the stable type comprising 51.73% and the high and sharp volatility types together makvolatile 83%. This suggests a significant presence of the stable type in low-grade FVC regions. Notably, regions with high CV values overlapped considerably with low-grade FVC areas, indicating that while vegetation in these areas improved, it became less stable and more fragile compared to other regions.

**Table 6:** Areas of the FVC stability type and its percentage.

CV	Volatility level	Area (%)
<0.07	Stabilization	17.09
0.07-0.17	Slight Fluctuation	47.37
0.17-0.30	Moderate Fluctuation	27.09
0.30-0.50	High Fluctuation	6.68
0.50-2.34	Wild Fluctuation	1.76

#### 3.1.3 Changes in the FVC of different classes

To quantitatively assess changes in FVC patterns, FVC class maps from different periods were superimposed to derive the FVC transfer matrix over time. Between 2001 and 2022, 11,130.56 km^2^ of various FVC grades experienced changes, excluding intra-grade transformations (**Table 7**). From 2001 to 2012 (**Table 8**), areas of medium and medium-high FVC increased, whereas low, medium-low, and high-grade FVC areas decreased. The low-grade FVC saw the largest decline, shrinking by 1,526.13 km^2^, primarily transitioning to medium-low (66.14%) and medium-grade FVC (26.94%). Medium-grade FVC exhibited the most significant area increase, totaling 1102.69 km^2^.

**Table 7:** Statistical data on the areas of different FVC grades from 2001 to 2022.

Year	Grade	2022					Total
		Low coverage	Medium-low coverage	Medium coverage	Medium-high coverage	High coverage	
2001	Low coverage	5257.19	1661.19	490.63	77.06	25.81	7511.88
	Medium-low coverage	412.69	1431.44	1360.63	427.13	90.69	3722.56
	Medium coverage	103.44	495.00	1416.75	1164.56	390.25	3570.00
	Medium-high coverage	37.94	130.19	940.31	1744.06	1368.63	4221.13
	High coverage	26.94	57.88	337.13	1252.81	4650.06	6324.81
	Total	5838.19	3775.69	4545.44	4665.63	6525.44	25350.38

**Table 8:** Statistical data on the areas of different FVC grades from 2001 to 2012.

Year	Grade	2012					Total
		Low coverage	Medium-low coverage	Medium coverage	Medium-high coverage	High coverage	
2001	Low coverage	5424.69	1380.38	562.25	122.94	21.63	7511.88
	Medium-low coverage	458.38	1307.31	1345.88	510.94	100.06	3722.56
	Medium coverage	81.13	552.50	1329.88	1165.50	441.00	3570.00
	Medium-high coverage	15.94	175.00	1041.56	1699.00	1289.63	4221.13
	High coverage	5.63	56.88	393.13	1410.25	4458.94	6324.81
	Total	5985.75	3472.06	4672.69	4908.63	6311.25	25350.38

**Figure 5:**
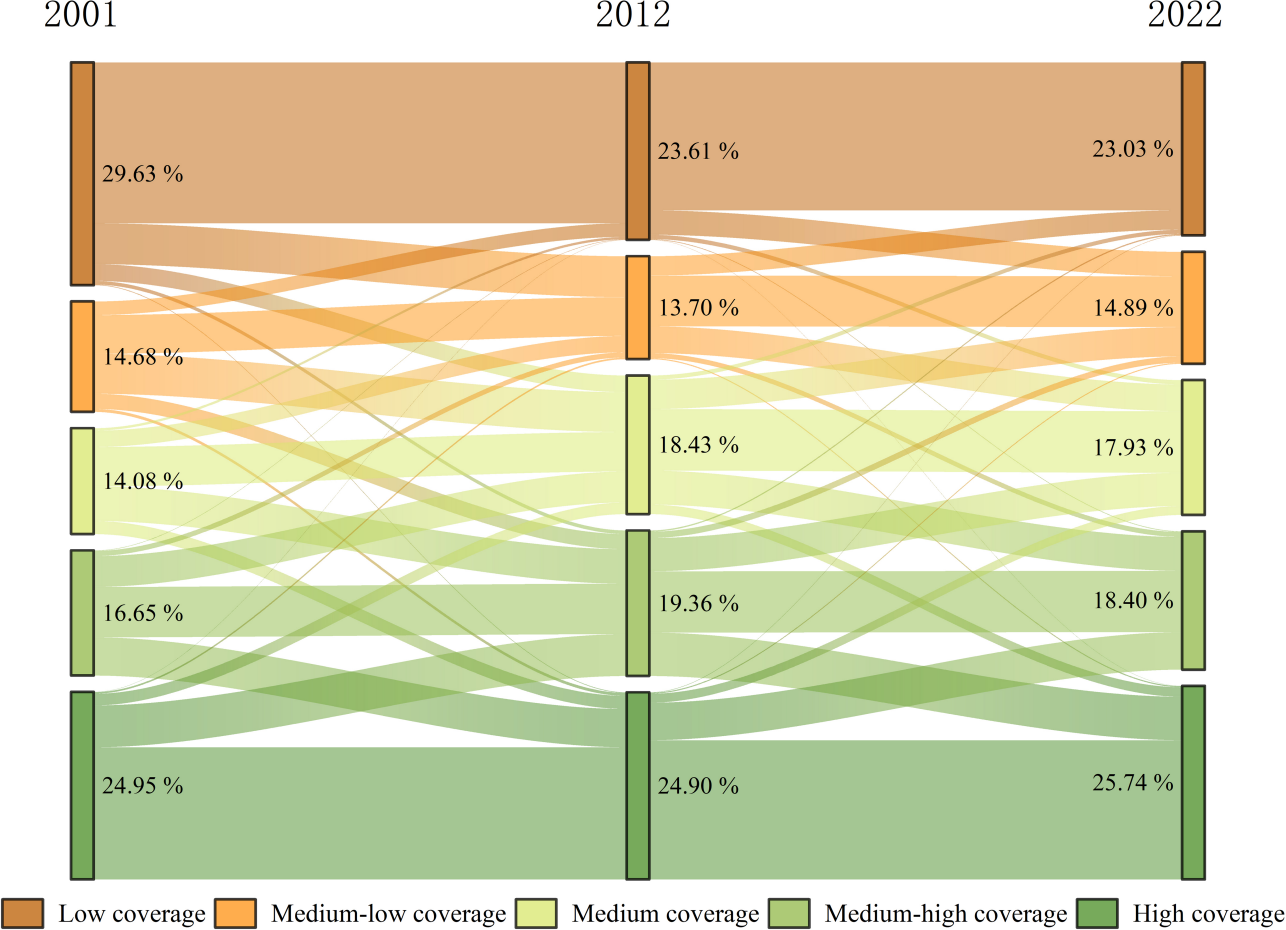
Sankey diagram of FVC transfer at each grade from 2001 to 2012 to 2022. (The width of the color band represents the proportion of different grades of FVC).

The expansion was primarily due to medium-low and medium-high FVC, comprising 40.26% and 31.16%, respectively. Between 2012 and 2022 (**Table 9**), FVC grade distribution shifted significantly. Medium-low and high-grade FVC areas increased, while low, medium, and medium-high FVC areas decreased. The largest area, 2599.38 km2, transferred to medium-high FVC, was predominantly medium and high FVC (44.02% and 48.63%, respectively). The high-grade FVC area grew mainly from medium and high-grade FVC, while medium and low-grade FVC areas increased primarily from medium and low-grade FVC (**Figure 5**). This suggests area conversion mainly occurs between adjacent FVC classes, with vegetation improvement shifting to higher FVC classes and degradation to lower ones. From 2001 to 2022, there was overall improvement and localized degradation, with vegetation improvement mainly in low to medium-grade FVC areas and degradation in medium to high-grade FVC areas.

**Table 9:** Statistical data on the areas of different FVC grades from 2012 to 2022.

Year	Grade	2022					Total
		Low coverage	Medium-low coverage	Medium coverage	Medium-high coverage	High coverage	
2012	Low coverage	4989.00	823.19	144.50	23.44	5.63	5985.75
	Medium-low coverage	652.81	1711.44	902.75	167.56	37.50	3472.06
	Medium coverage	139.75	991.88	2075.94	1144.25	320.88	4672.69
	Medium-high coverage	43.25	212.94	1121.31	2066.25	1464.88	4908.63
	High coverage	13.38	36.25	300.94	1264.13	4696.56	6311.25
	Total	5838.19	3775.69	4545.44	4665.63	6525.44	25350.38

### 3.2 Topographic effects of the FVC

Topography is a crucial nonzonal factor influencing vegetation growth by altering the spatial distribution through its impact on air temperature, precipitation, and soil characteristics. This study examines how elevation, slope, and slope orientation affect changes in the FVC. FVC is categorized into three types: degraded, stabilized, and improved. Elevation is divided into four classes using the natural breakpoint method (Coulson, 1987): low-elevation (<3500m), middle-low elevation (3500–3800m), middle-elevation (3800–4200m), and high-elevation (>4200m). Slope direction is classified into five categories (Han et al., 2023): flat (-1°), shaded (0-67.5°, 337-360°), semishaded (67.5-112.5°, 292.5-337.5°), semipositive (112.5-157.5°, 247.5-292.5°), and sunny (157.5-247.5°). Slopes are also categorized into five classes according to the natural breakpoint method (Coulson, 1987): flat (0-3°), gently sloping (3-7°), sloping (7-12°), steeply sloping (12-18°), and sharply sloping (>18°).

#### 3.2.1 Distributional characteristics of FVC change types at different elevations

**Figure 6:**
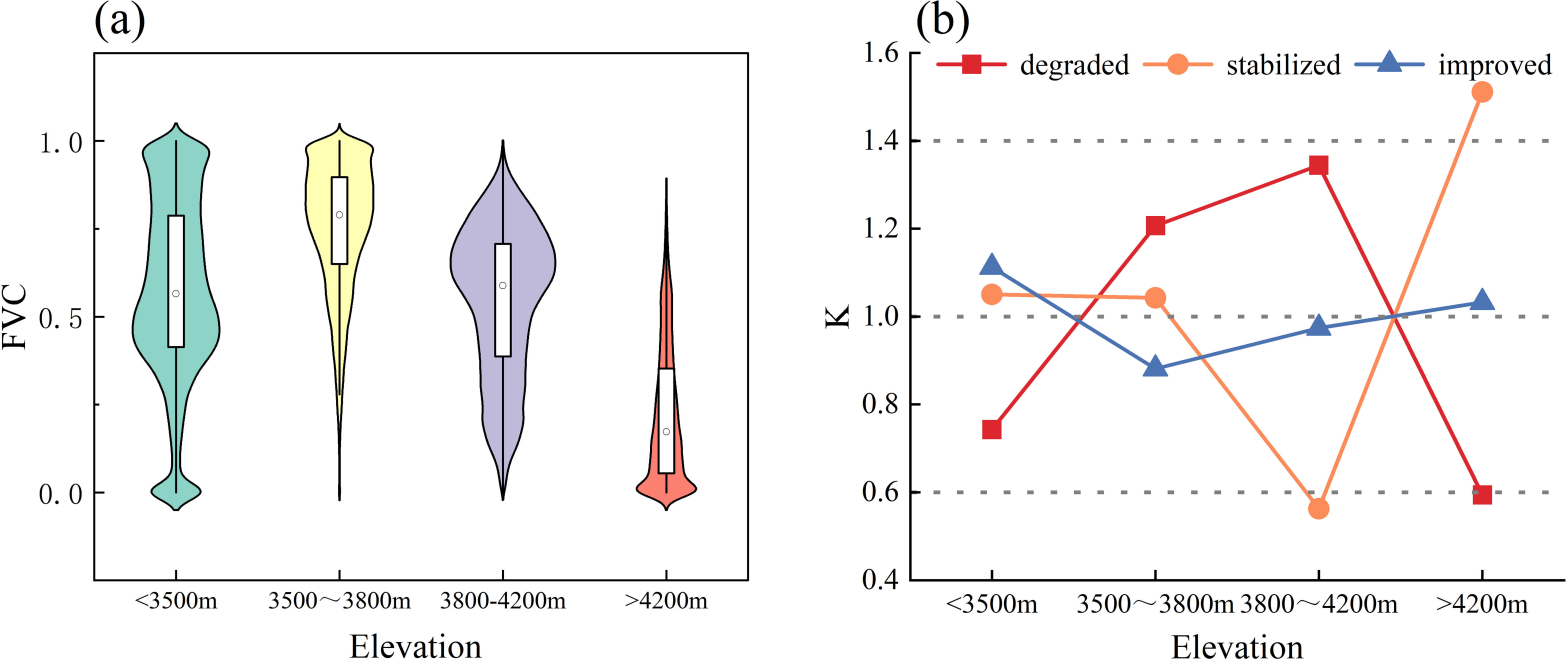
The topographic effects of FVC at different elevation gradients. **(A)** Distribution of FVC under different altitude intervals. **(B)** Distribution dominance of FVC change types at different altitude gradients, K-values is the Terrain distribution index, If k>1, it indicates that type i changes are dominantly distributed in terrain e; if k=1, it indicates that type i changes are evenly distributed in terrain e; if k<1, it indicates that type i changes are not dominantly distributed in terrain e.

The changes in the FVC at different elevations (**Figure 6A**) were concentrated between 0.40-0.50 at low elevations, between 0.6 and 0.8 at middle-low and middle elevations, and an important concentration of FVC between 0.00 and 0.20 at high elevation. From the viewpoint of vegetation change type, the change in FVC at different elevations in the Qinghai Lake Basin varied significantly, and in general, the K values of the vegetation stabilization and vegetation stabilization types at different elevations clearly tended to decrease first and then increase, and the K values of the vegetation degradation types tended to increase first and then decrease (**Figure 6B**). Specifically, the vegetation degradation type is distributed mainly at middle-low and middle elevations. The vegetation stabilization type is distributed mainly in low-, middle-low- and high-elevation areas, and the K value in high-elevation areas is the highest, at 1.51. The vegetation improvement type is distributed mainly in low-elevation areas, and the vegetation stabilization type is distributed mainly in high-elevation areas.

#### 3.2.2 Distributional characteristics of FVC change types at different slopes

**Figure 7:**
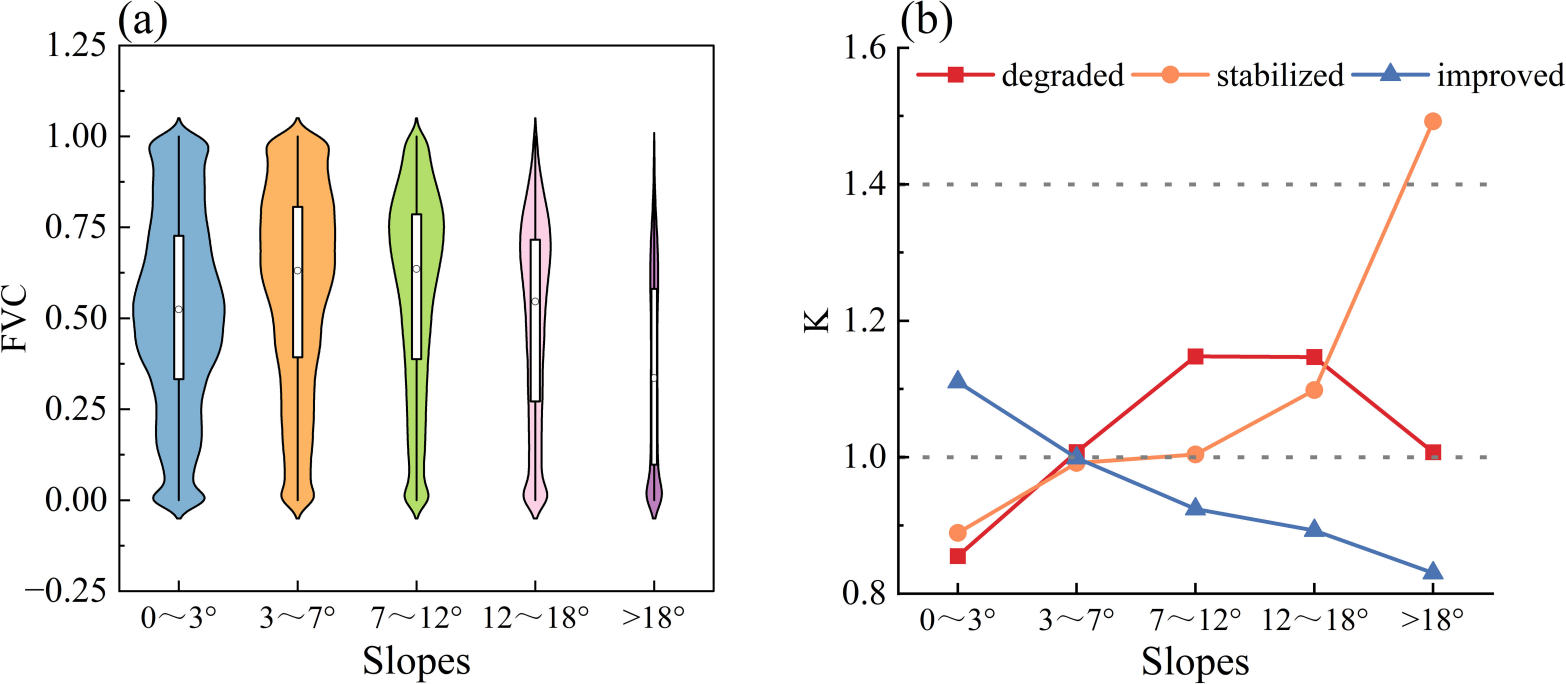
The topographic effects of FVC at different slopes. **(A)** Distribution of FVC under different slopes. **(B)** Distribution dominance of FVC change types at different slopes, K-values is the Terrain distribution index, If k>1, it indicates that type i changes are dominantly distributed in terrain e; if k=1, it indicates that type i changes are evenly distributed in terrain e; if k<1, it indicates that type i changes are not dominantly distributed in terrain e.

The distribution of the FVC became increasingly discrete with increasing slope, concentrating between 0.40 and 0.60 within a slope of 0-3°, which is mainly concentrated between 0.60 and 0.80 within the interval of 3°-20°and is mainly concentrated between 0.00 and 0.10 within the interval of greater than 20° (**Figure 7A**). The vegetation-improving type was dominant mainly on flat slopes; the vegetation-degrading type was dominant mainly on gently sloping, sloping and steep slopes; and the vegetation-stabilizing type was dominant mainly on sharply sloping land (**Figure 7B**), indicating that vegetation growth was unfavorable with increasing slope gradient.

#### 3.2.3 Distributional characteristics of FVC change types with different slope directions

**Figure 8:**
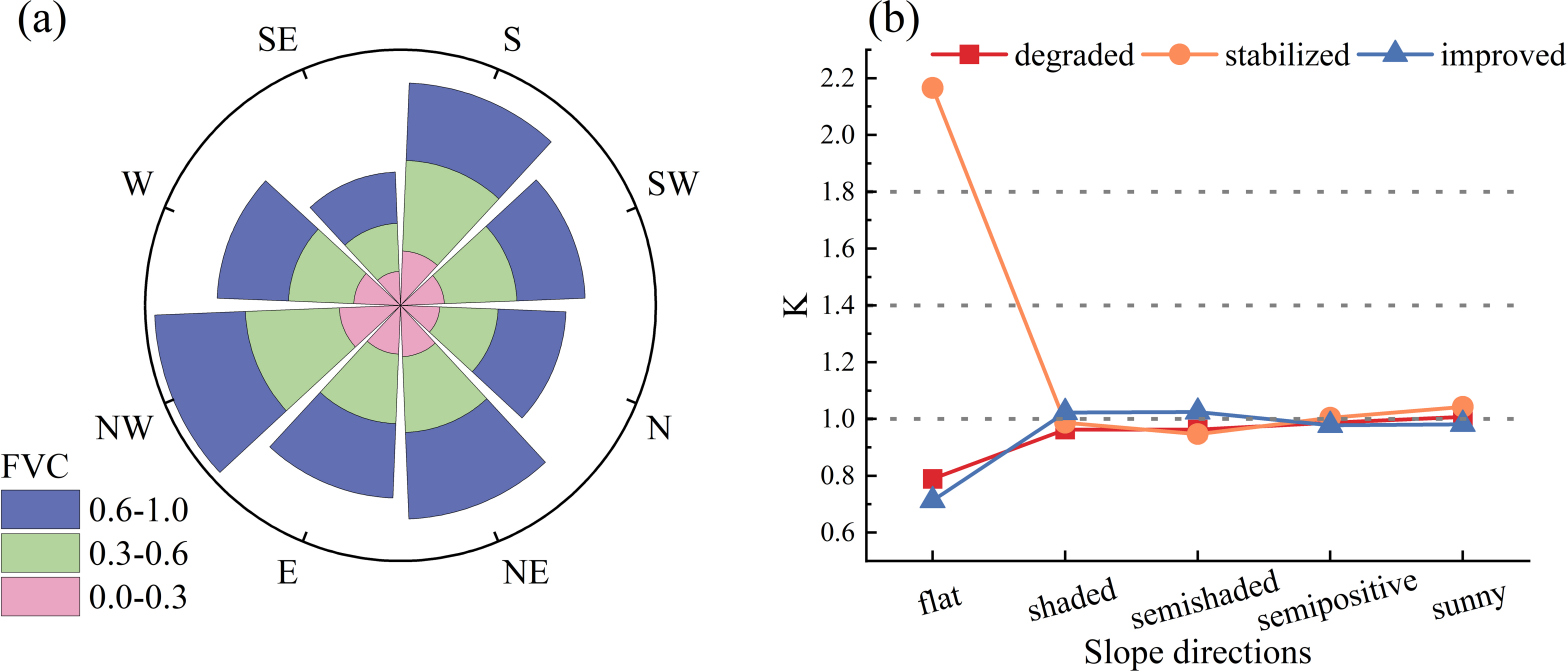
The topographic effects of FVC at different slope directions. **(A)** Distribution of FVC under different slope directions. **(B)** Distribution dominance of FVC change types at different slope directions, K-values is the Terrain distribution index, if k>1, it indicates that type changes are dominantly distributed in terrain; if k=1, it indicates that type changes are evenly distributed in terrain; if k<1, it indicates that type changes are not dominantly distributed in terrain.

The distribution of the FVC became increasingly discrete with increasing slope, concentrating between 0.40 and 0.60 within a slope of 0-3°, which is mainly concentrated between 0.60 and 0.80 within the interval of 3°-20°and is mainly concentrated between 0.00 and 0.10 within the interval of greater than 20° (**Figure 8A**). The vegetation-improving type was dominant mainly on flat slopes; the vegetation-degrading type was dominant mainly on gently sloping, sloping and steep slopes; and the vegetation-stabilizing type was dominant mainly on sharply sloping land (**Figure 8B**), indicating that vegetation growth was unfavorable with increasing slope gradient.

### 3.3 Analysis of drivers of FVC

#### 3.3.1 Influence of driving factors on the spatial distribution patterns of the FVC

The spatial distribution pattern of the Fractional Vegetation Cover (FVC) was investigated by examining the influence of various environmental and anthropogenic factors. The average FVC from 2001 to 2022 was used as the dependent variable, while surface factors (elevation, slope, aspect, soil type, and soil organic carbon content), climatic factors (air temperature and precipitation), and human activity factors (land use, population density, and grazing intensity) were considered as potential driving forces. The 22-year average values of each factor were calculated and analyzed using a geodetector approach. The results showed that the P-values for all individual drivers and their interactions were less than 0.05, indicating that the spatial distribution pattern of the FVC can be comprehensively explained by the combined effects of these factors.

**Figure 9:**
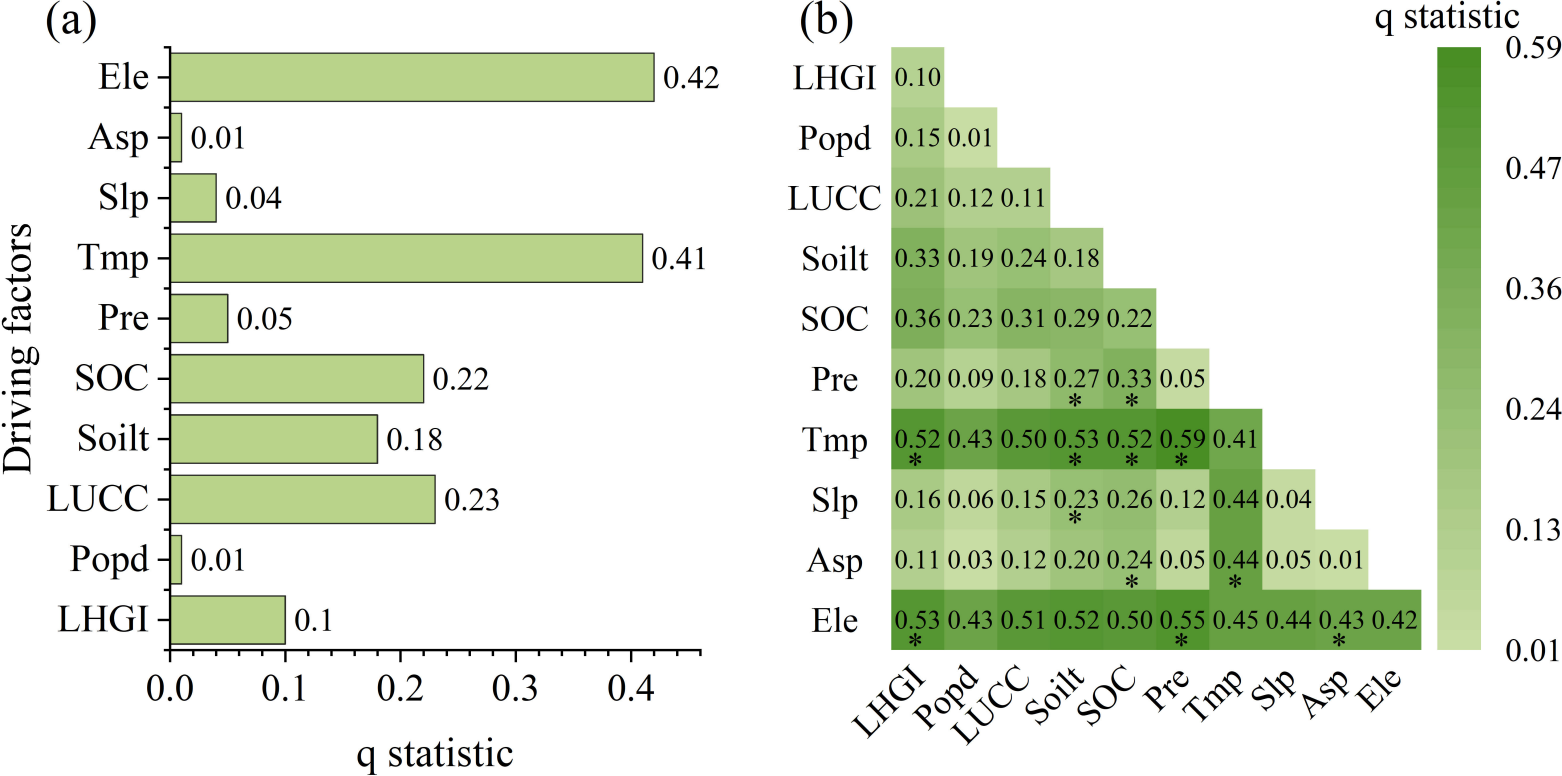
Factor detection and interaction detection results of the drivers of the FVC. **(A)** Driver factors and their q values. **(B)** Driver interactions and their q values. (Asp-Slope direction, Slp-Slope, Ele-Elevation, Tmp-Temperature, Pre-Precipitation, LUCC-Land cover, LHGI-long-term High resolution Grazing Intensity, Popd-Population density, Soilt-Soil type, SOC-Soil organic carbon). The ‘*’ symbol denotes nonlinear enhanced type, with all others being bivariable enhanced.

The relative importance of the drivers in explaining the observed changes in fractional vegetation cover (FVC) was assessed through a hierarchical analysis. The drivers were ranked in descending order of explanatory power as follows: elevation > temperature > land use > grasslands grazing intensity > soil organic carbon content > soil type > precipitation > slope > population density > slope orientation (**Figure 9A**). Notably, elevation and temperature had the greatest individual effects, with the other factors exhibiting smaller, yet significant, interactive influences. The interaction detection analysis revealed the complex, nonlinear relationships between the drivers and FVC changes (**Figure 9B**). The two-factor interaction effects, as indicated by the q-values, were generally stronger than the individual driver effects. This suggests synergistic and amplifying interactions among the drivers. Particularly strong interactions (q>0.40) were observed between air temperature and other factors, as well as between elevation and the remaining drivers. The interaction between temperature and precipitation was the most pronounced (q=0.59). Additionally, there were strong interactions (q>0.50) between temperature and topography, land use, soil type, and soil organic carbon content.

Factor detection quantified the explanatory power of different drivers on the spatial distribution of FVC, the factors ranked from highest to lowest explanatory power (q-value) were elevation > temperature > land use > grasslands grazing intensity > soil organic carbon content > soil type > precipitation > slope > population density > slope orientation (**Figure 9A**). The interaction detector analyzed the complex effects of complex interactions among drivers on FVC. Results revealed that all interactions exhibited both bivariate and nonlinear enhancement effects. Particularly strong interactions (q>0.40) were observed between air temperature and other factors, as well as between elevation and the remaining drivers. The interaction between air temperature and precipitation was the strongest (q=0.59), indicating an enhancing effect of their interaction on FVC. Additionally, the interactions between air temperature and soil type, as well as between air temperature and soil organic carbon content, were also prominent, suggesting synergistic effects between air temperature and soil factors that jointly influence vegetation cover.

#### 3.3.2 Exploration of the driving mechanism via the PLS–SEM

To examine the direct and indirect influences of various drivers on FVC, the cumulative impact of these drivers on FVC is the combined sum of their direct and indirect effects. Through the utilization of Partial Least Squares Structural Equation Modeling (PLS-SEM), a more comprehensive elucidation of the potential influences and interrelations among the drivers can be achieved by incorporating latent variables. Owing to their low q values in the geodetector analysis of the drivers’ impacts on the spatial distribution of FVC, slope orientation and population density were excluded from the model (MA et al., 2024a). Four sets of latent variables were established: climatic factors (temperature and precipitation), topographical factors (elevation and slope), soil characteristics (soil type and organic carbon content), and human-related factors (grazing intensity and land use). Prior to constructing the PLS-SEM model, the variance inflation factor (VIF) was employed to assess multicollinearity among the explanatory variables, evaluating the degree of collinearity between them. The results indicated that all VIF values were below 5 (**Table 10**), suggesting no significant covariance existed between the variables (Thompson et al., 2017; Wang et al., 2022a). Convergent validity was verified using average variance extracted (AVE), with all latent variables exhibiting AVE values exceeding 5 (Nasution et al., 2020). This confirms that all factor loadings meet the requirements for structural validity (**Table 11**). The model performance metrics are detailed in **Table 10**. The R² value indicates the model’s strong explanatory power, reflecting its effectiveness in predicting endogenous latent variables. The Goodness-of-Fit (GOF) value demonstrates the model’s robust fit. Furthermore, all p-values are below 0.05, indicating that the path coefficients are statistically significant. These results underscore the robust explanatory capacity of PLS-SEM in delineating causal pathways (**Table 3**).

**Table 10:** Variance inflation factor (VIF) for each observed variable in the PLS-SEM model.

Latent variables	Observed variables	VIF			
		2001-2022	2001	2012	2022
Climate	Temperature (Tmp)	3.54	3.58	3.43	3.70
	Precipitation (Pre)	1.36	1.38	1.35	1.47
Top	Elevation (Ele)	3.80	3.85	3.67	3.89
	Slope (Slp)	1.43	1.42	1.42	1.42
SOC	Soil type (Soilt)	1.34	1.34	1.34	1.34
	Soil organic carbon (SOC)	1.31	1.32	1.31	1.30
Human	land-use change (LUCC)	1.44	1.02	1.01	1.02
	Grazing intensity (LHGI)	1.40	1.31	1.38	1.43

**Table 11:** Model performance of the PLS-SEM.

Model performance indicator	Types	Values			
		2001-2022	2001	2012	2022
R^2^	Climate	0.77	0.52	0.50	0.50
	Top	0.65	0.61	0.51	0.60
	Human	0.72	0.71	0.81	0.75
	Soil	0.23	0.21	0.21	0.21
AVE	Climate	0.52	0.62	0.51	0.62
	Top	0.73	0.72	0.73	0.72
	Human	0.45	0.51	0.48	0.50
	Soil	0.69	0.70	0.68	0.69
GOF	/	0.47	0.42	0.42	0.43

**Figure 10:**
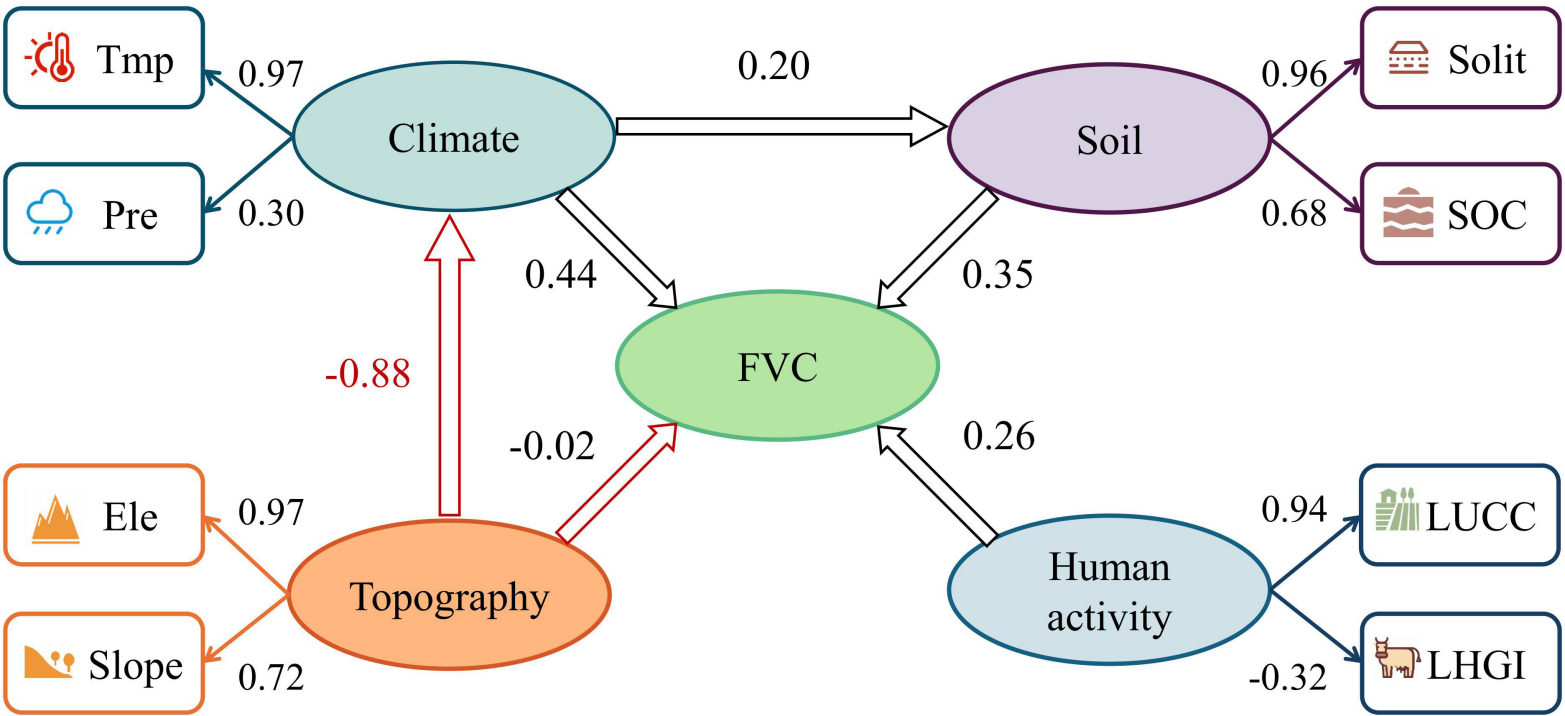
The PLS-SEM model results illustrate the pathways and degrees of influence exerted by climatic factors (temperature(Tmp) and precipitation(Pre)), topographical factors (elevation(Ele) and slope(Slp)), soil factors (soil type(Solit) and soil organic carbon(SOC)), and anthropogenic factors (land-use change(LUCC) and grazing intensity(LHGI)) on FVC. Ellipses denote latent variables, while rectangles represent observed variables. Arrows indicate associations between them. Red signifies negative correlations; black denotes positive correlations.

The total effect values of latent variables on FVC were ranked as follows (**Table 12**): climatic variables (0.46) > topographic variables (-0.46) > soil variables (0.35) > anthropogenic variables (0.26). Among the direct effects on FVC (**Figure 10**), climatic (0.44) and soil variables (0.35) were predominant. Conversely, the primary effect was the inhibitory influence of topography on FVC, which negatively moderated the climatic variable (-0.42), while the climatic variable also contributed to FVC by affecting the soil factor (0.07).

**Table 12:** Direct, indirect and total impact of latent variables on FVC.

Relationship	Direct	Indirect	Total
Top→ FVC	-0.02	-0.44	-0.46
Climate → FVC	0.44	0.07	0.50
Human → FVC	0.26	0.00	0.26

**Figure 11:**
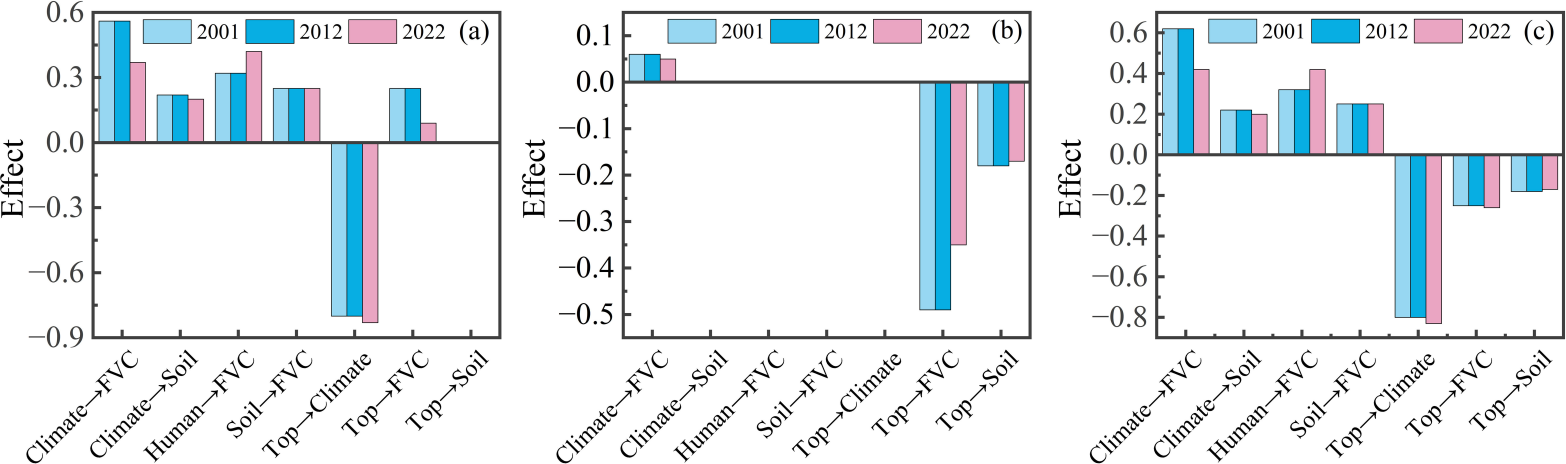
Impact of each latent variable on the FVC by PLS-SEM (2001, 2012 and 2022). **(a) **Direct impact **(B)** Indirect impact **(C)** Total impact.

To assess the drivers influencing FVC dynamics, models for 2001, 2012, and 2022 were developed to quantify each driver’s direct and indirect effects on FVC changes (**Figure 11**). Climate variables exerted the greatest overall influence on FVC, though their direct impact diminished over time (0.60, 0.47, 0.35). The direct negative effects of anthropogenic factors initially increased but subsequently decreased (-0.06, -0.19, -0.13). Topographic factors shifted from a facilitating role in 2001 to an inhibitory one in 2012 and 2022 (0.12, -0.02, -0.07), with their overall effect remaining negative (-0.36, -0.43, -0.36). Soil factors consistently contributed to a stable positive effect on FVC (0.31, 0.33, 0.32).

## 4 Discussion

### 4.1 Main mechanisms affecting the spatial distribution of the FVC in the Qinghai Lake Basin

Geodetector results indicate that both temperature and elevation exert greater spatial explanatory power over FVC than other factors, whether acting as single drivers or through dual interactions. Among these, the interaction between temperature and precipitation exerts the most significant influence on FVC. This demonstrates that climatic elements do not operate in isolation but rather affect FVC through synergistic interactions. Furthermore, PLS-SEM model results quantitatively rank the importance of each driving factor (climatic variables > topographic variables > soil variables > anthropogenic variables). Among climatic variables, the loading of temperature (0.97) substantially exceeded that of precipitation (0.30); among topographic variables, the loading of elevation (0.97) surpassed that of slope (0.72). This confirms the positive effect of climate warming on vegetation in mid-to-high and high-altitude regions of northern latitudes (Wei et al., 2023), where climatic conditions dominate and constrain the formation of vegetation cover spatial patterns at the macro level (Woodward and McKee, 1991).

**Figure 12:**
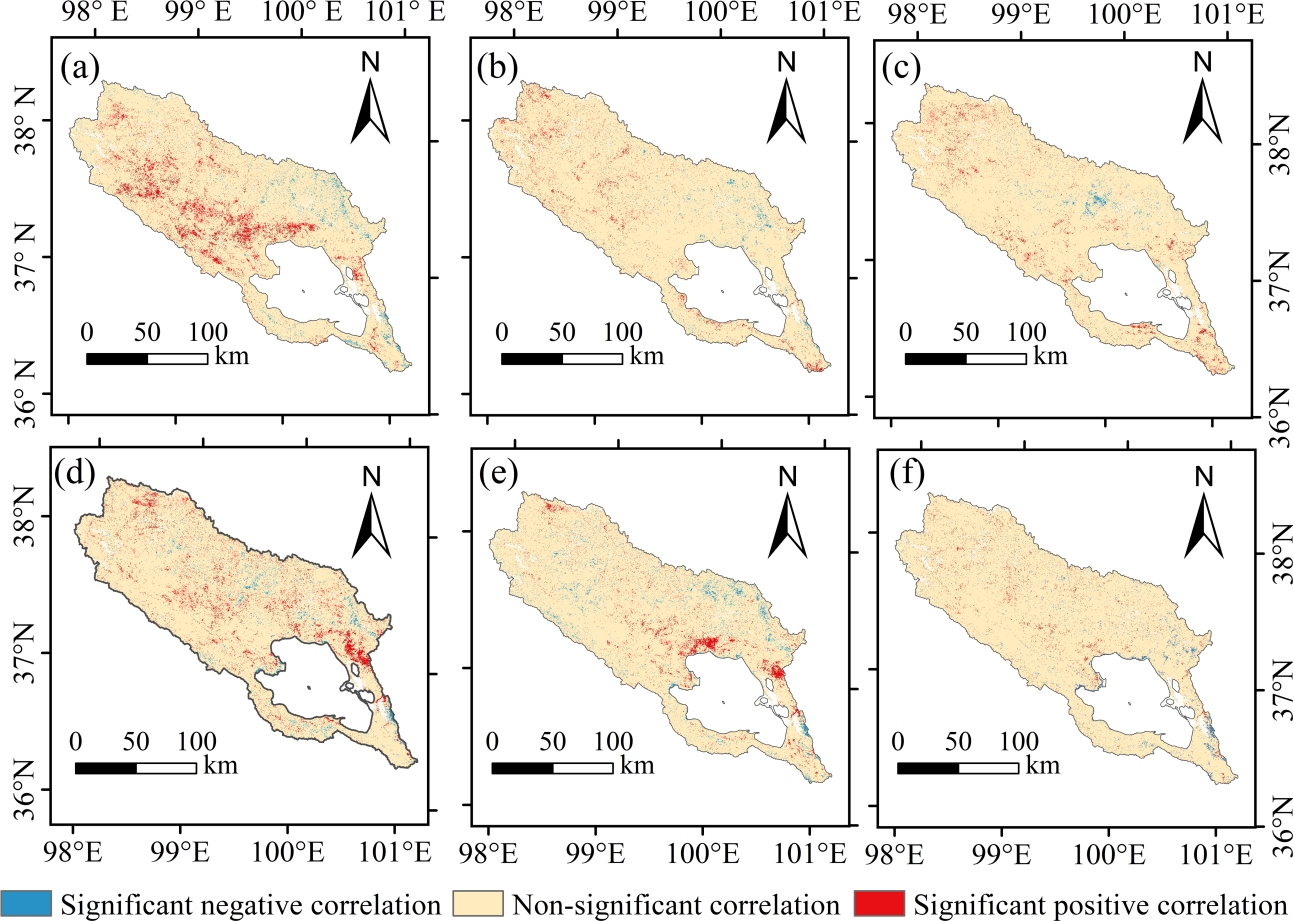
Correlations between precipitation at different lag periods and temperature and FVC. **(A–C)** represent the correlation between precipitation and FVC at lags of 0-year, 1-year, and 2-year respectively; **(D**–**F)** represent the correlation between temperature and FVC at lags of 0-year, 1-year, and 2-year respectively.

Path analysis indicates that, on the one hand, climatic variables exert a direct promoting effect on FVC (**Figure 10**). **Figure 12** depicts how the correlation between temperature and precipitation varies with annual lags of 0, 1, and 2 years, highlighting the dependence of their association on temporal delays. and FVC. Spatially, precipitation exhibits no significant lagged effect on FVC, whereas temperature shows locally significant correlations with FVC only at lag 1. During the same period, regions exhibiting a positive response of FVC to temperature were primarily distributed in the northeastern area of Qinghai Lake and the high-altitude regions in the central and northwestern parts of the basin. The extensive distribution of alpine steppe grasses, such as Stipa purpurea, Kobresia pygmaea, and Sibbaldia tetrandra, whose photosynthetic enzyme activity is more sensitive to temperature, contributes to this pattern (Xiang et al., 2021).

Moderate warming enhances the activity of photosynthetic enzymes in vegetation (Moore et al., 2021), improves photosynthetic efficiency, and promotes plant growth and development. Furthermore, temperature increases mitigate low-temperature stress in high-altitude regions to some extent, thereby facilitating vegetation improvement (Wang et al., 2023c). The regions exhibiting a positive response of FVC to precipitation are primarily distributed in the middle reaches of the watershed and the northwestern area of Qinghai Lake, with partial coverage in the upper reaches of the Buha River (**Figure 12A**). In low-to-medium elevation zones, suitable precipitation conditions foster abundant soil moisture, thereby supporting vegetation growth (Dai et al., 2022).

**Figure 13:**
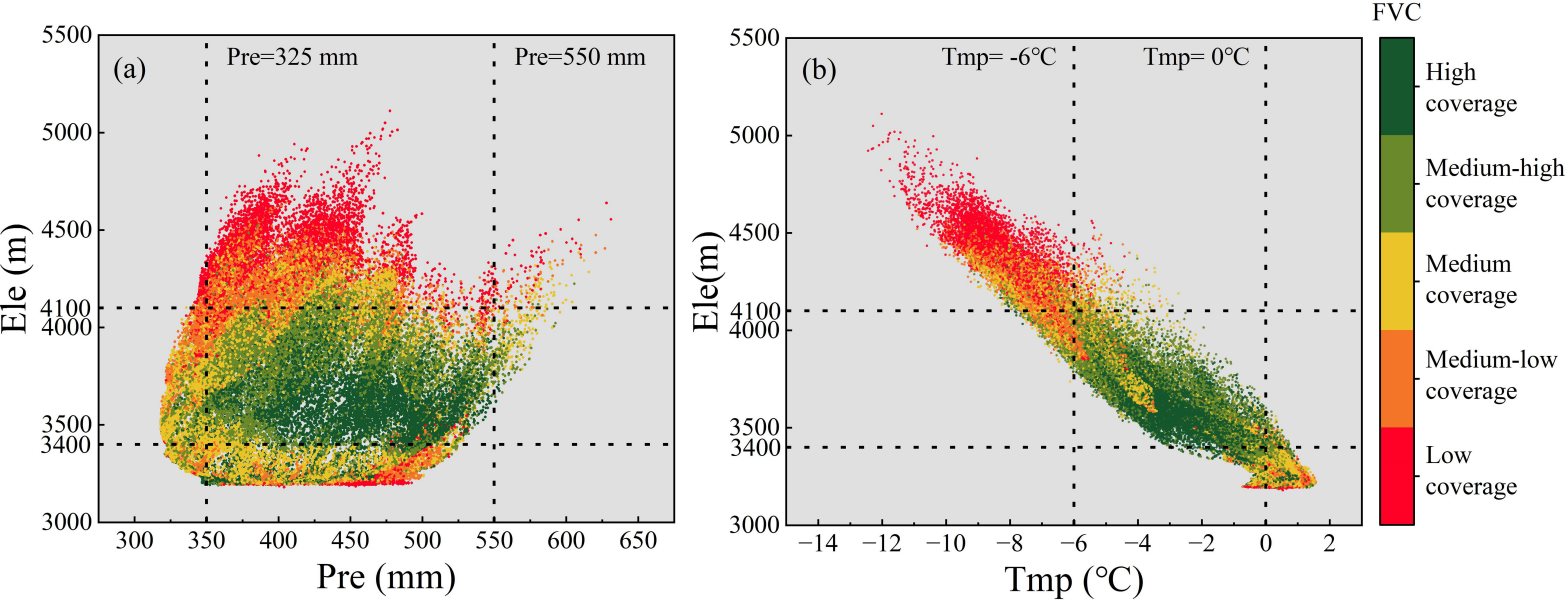
Relationship between FVC and climatic factors (temperature and precipitation) at different elevations. **(A)** Annual mean total precipitation and FVC. **(B)** Annual mean air temperature and FVC.

On the other hand, within the process whereby topographical factors indirectly influence FVC, elevation exerts a crucial regulatory effect on both air temperature and precipitation. **Figure 13** illustrates the relationship between temperature, precipitation, and FVC at different elevations, revealing that vegetation exhibits a non-linear response to both temperature and precipitation (Knapp et al., 2017). Determine the optimal range based on the distribution of high-grade and medium-to-high-grade FVC. Specifically, when precipitation falls below 325 mm, arid conditions constrain the water supply required for vegetation growth, inhibiting photosynthesis and leading to reduced vegetation cover (Tezara et al., 1999). Conversely, when precipitation exceeds 550 mm, excessive rainfall impairs soil aeration, causing root hypoxia that suppresses plant respiration and growth (Ben-Noah and Friedman, 2018).

Temperature and elevation exhibit a stronger linear dependency (Piao et al., 2011; Tao et al., 2014, 2015). When temperatures fall below -6°C, the extreme cold conditions at high elevation impair vegetation’s water uptake efficiency, inhibiting growth (Zhai et al., 2024). Conversely, temperatures above 0°C induce heat stress that accelerates soil nutrient leaching, leading to a sharp decline in vegetation organic matter (Fissore et al., 2008). Furthermore, it is noteworthy that within the 3400–4100 m elevation range, FVC exhibits heightened sensitivity to temperature fluctuations. Even within the optimal temperature range, FVC struggles to attain high values at greater elevations. Moreover, when temperatures deviate from the optimal range, the decline in FVC becomes markedly more pronounced. This evidence further demonstrates how elevation modulates temperature’s influence on FVC by altering hydrothermal conditions (Guo et al., 2023).

Vegetation cover in the Qinghai Lake basin is primarily driven by a synergistic mechanism of “climate dominance, topography regulation”. Regarding climatic factors, temperature and precipitation do not exert isolated effects on FVC but influence it through synergistic interactions, with temperature demonstrating a more pronounced driving role. Elevation, as the core topographic factor, indirectly regulates the influence of climatic factors on FVC by altering the spatial distribution of water and heat resources. Concurrently, elevation and hydrotemperate conditions exhibit threshold effects: within the mid-to-low elevation range of 3400–4100 m, FVC responds more sensitively to hydrotemperate conditions. Conversely, in high-elevation areas constrained by extreme environments, FVC is generally lower. Precipitation (325–550 mm) and air temperature (-6-0°C) exhibit optimal ranges for vegetation growth; exceeding these thresholds inhibits FVC.

### 4.2 The main factors influencing the change in the FVC in the Qinghai Lake Basin at the time scale

**Figure 11** indicates that climatic variables primarily drive temporal variations in forest cover within the Qinghai Lake watershed. From 2001 to 2022, the direct positive influence of climatic variables on forest cover diminished, while the positive impact of human activities intensified. Spatio-temporal analysis of FVC indicates that vegetation degradation is concentrated in the central basin and northwestern Qinghai Lake regions. The trend towards climate warming and moistening has intensified evapotranspiration (Cui et al., 2019; Liu et al., 2021c), thereby reducing regional water replenishment from precipitation (Liu et al., 2021b; Zhou and Yu, 2025). As previously noted, when climatic conditions exceed the upper limit for vegetation growth, they conversely constrain vegetation improvement.Given the heightened sensitivity of vegetation in low-medium elevation regions, areas experiencing vegetation degradation within the middle reaches of the watershed may be planted with shrubs possessing strong soil-stabilising capabilities to prevent soil erosion (Yu et al., 2022). For the northwestern region of Qinghai Lake, measures may include applying organic fertilisers prior to the vegetation regrowth season to enhance soil fertility, alongside implementing seasonal grazing practices to prevent damage to vegetation recovery on steep slopes (Wang et al., 2020).

The intensified direct promotion of human activities on FVC suggests that recent efforts, such as the creation of ecological protection zones and policies to revert grazing land to grassland (Shao et al., 2017; Yan et al., 2022), have facilitated grassland vegetation restoration. Owing to the intensification of overgrazing in the late 1990s, which peaked in 2000 (Wu et al., 2021), the ecological environment in the northwestern part of the Tibetan Plateau was severely damaged, inhibiting vegetation growth in the ecologically fragile Qinghai Lake Basin. However, existing research has demonstrated that the policies and measures implemented after 2000, such as grazing bans (Yao et al., 2018) and the establishment of nature reserves (Shao et al., 2017; Liu et al., 2019a), contributed to vegetation recovery.

## 5 Conclusion

This study analyzed the spatiotemporal evolution characteristics of the FVC and its topographical effects in the largest internal drainage basin (Qinghai Lake Basin) of the Qinghai–Tibet Plateau from 2001 to 2022. The authors also used geodetectors and PLS-SEMs to investigate the effects of different driving factors on the FVC distribution and discussed the driving mechanisms of spatiotemporal changes in the FVC. The conclusions of the study are as follows:

(1) From 2001–2022, the FVC in the Qinghai Lake Basin showed a significant fluctuating growth trend. Spatially, the FVC in the Qinghai Lake Basin exhibited a pattern of overall improvement and localized degradation. Areas of significant improvement were primarily concentrated in the northwestern region of the basin and the Habu River Basin, where FVC levels primarily transitioned from low-grade FVC and medium-low FVC to medium FVC and medium-high FVC. Areas of significant degradation were primarily concentrated in the northern and central regions of the basin, where FVC levels primarily transitioned from high FVC and medium-high FVC to medium-grade FVC. The basin’s FVC exhibits low spatial variability overall, and areas with low-grade FVC and high variability highly overlap.

(2) Under different terrain conditions, the FVC in the Qinghai Lake Basin first increases but then decreases with increasing elevation, with the average FVC being highest at lower elevations. The distribution of the FVC values becomes increasingly dispersed as the slope steepness increases. The FVC values of the semishaded slopes of the Qinghai Lake Basin are the most concentrated, followed by those of the shaded slopes, semisunny slopes, and sunny slopes. Additionally, the distribution of vegetation change types varies significantly under different terrain conditions: vegetation improvement types are primarily distributed at low elevations, flat slopes, and shaded and semishaded slopes; vegetation degradation types are primarily distributed at mid-elevations, gentle to steep slopes, and sunny slopes; and vegetation stability types are primarily distributed at high-elevations, steep slopes, and semisunny slopes.

(3) In the single-factor analysis, the driving factors were ranked by explanatory power as follows: elevation>temperature>land use>soil organic carbon content>soil type>precipitation>slope>population density>slope aspect. From the interaction analysis, the interactions between temperature and all other factors, as well as the interactions between elevation and all other factors, are all above 0. 40. The total effect values of the latent variables on the FVC, ranked from highest to lowest, are as follows: climate variables>topographic>variables>soil variables>human-induced variables. Climate factors exert a direct positive effect on FVC. Temperature and precipitation jointly influence FVC through synergistic effects, with temperature playing a more significant driving role. Topography primarily influences FVC indirectly by regulating hydrological and thermal conditions (temperature and precipitation). Each factor exhibits an optimal range (elevation: 3400–4100 m, precipitation: 325–550 mm, temperature: −6 to 0°C). When driving factors exceed these optimal ranges, FVC is suppressed. Climate and human factors are the main contributors to the temporal changes in the FVC in the Qinghai Lake Basin. Under the influence of policies such as the establishment of nature reserves and grazing bans, the positive role of human factors in the temporal changes in the FVC is increasing, indicating that reasonable human activities and appropriate management measures can contribute to the recovery and protection of regional ecosystems.

## Data Availability

The original contributions presented in the study are included in the article/supplementary material. Further inquiries can be directed to the corresponding author.
